# Propofol Attenuates Brain Ischemic Injury Through the Cannabinoid Type 1 (CB1) Receptor

**DOI:** 10.7759/cureus.106641

**Published:** 2026-04-08

**Authors:** Ji Jia, Ming Cao, Yuanyuan Zhang, Jing Lang, Rui Xiao, Jie Peng, Bo Xu

**Affiliations:** 1 Anesthesiology, General Hospital of Southern Theatre Command, Guangzhou, CHN; 2 Geriatrics, General Hospital of Southern Theatre Command, Guangzhou, CHN; 3 Orthopedics, General Hospital of Western Theatre Command, Chengdu, CHN

**Keywords:** brain ischemic injury, cannabinoids type 1 receptor, oxidative stress injury, propofol, rat

## Abstract

Background and objective

Although the anesthetic propofol has been shown to reduce ischemic brain injury, its precise neuroprotective mechanisms remain unclear. Many studies have reported that propofol injection can increase endocannabinoid levels in vivo and in vitro. This study investigates the cerebroprotective pathways mediated by propofol during ischemic insults.

Methods

In vivo studies used a rat middle cerebral artery occlusion (MCAO) model to simulate stroke-related cerebral ischemia/reperfusion injury. In vitro, oxygen-glucose deprivation/reoxygenation (OGD/R) models were used to mimic ischemic neuronal conditions in primary cultured neurons. Propofol was used to treat rats with MCAO and neurons receiving OGD/R injury. After the treatments, injury levels in rats and neurons were evaluated.

Results

In animal models, treatment with propofol significantly reduced cerebral infarction volume, improved neurological outcomes, decreased neuronal apoptosis, and lowered oxidative stress markers, including NOX2 gp91 subunit expression and malondialdehyde concentrations, compared to the untreated MCAO controls (P < 0.05). Concurrently, propofol administration elevated antioxidant defenses by enhancing superoxide dismutase 2 activity and glutathione production. Cellular experiments demonstrated propofol's ability to preserve membrane integrity (reduced lactate dehydrogenase leakage), decrease apoptotic signaling, and restore redox balance by modulating reactive oxygen species (ROS) generation and antioxidant reserves (P < 0.05). Pharmacological intervention revealed that cannabinoid type 1 (CB1) receptor blockade, but not cannabinoid type 2 receptor antagonism (P > 0.05), effectively negated propofol’s protective effects in both experimental models. Biochemical analyses identified that endogenous cannabinoids (anandamide (AEA) and 2-arachidonoylglycerol (2-AG)) were increased in the serum following propofol administration in ischemic subjects.

Conclusions

The findings of this investigation demonstrate that propofol induces neuroprotection and produces endocannabinoids (AEA and 2-AG) in vivo and in vitro. In vivo, propofol protects ischemic brain tissue in rats and reduces oxidative stress. In vitro, propofol reduces MCAO-induced neuronal injury by decreasing ROS generation and restoring antioxidant levels. However, the CB1 receptor antagonist abolishes propofol’s protective effects in vivo and in vitro, indicating that the CB1 receptor mediates propofol’s neuroprotective activity against ischemic cerebral damage.

## Introduction

Ischemic stroke remains a major global public health concern, with significant epidemiological disparities between nations. In 2019, China reported 3.94 million new stroke cases compared to 795,000 in the United States, where 84.1% of the cases were ischemic in nature [1]. Current therapeutic strategies face substantial limitations, as recombinant tissue-type plasminogen activator (r-TPA) remains the sole validated treatment despite its restrictive 4.5-hour therapeutic window [2]. This temporal constraint contributes to China’s notably low thrombolysis rate of 3.2% (2019-2020), underscoring the critical need for novel therapeutic interventions [2].

The pathophysiology of cerebral ischemia involves a complex cascade, including neuronal apoptosis, inflammatory responses, and oxidative stress [3-5]. Paradoxically, while r-TPA-mediated reperfusion restores cerebral circulation, it concurrently exacerbates oxidative damage through reactive oxygen species (ROS) generation [6], which overwhelms endogenous antioxidant defenses and potentiates neuronal membrane degradation [7-9]. These mechanisms highlight the therapeutic potential of antioxidant strategies in stroke management.

Propofol, a widely utilized IV anesthetic, has demonstrated antioxidant properties in cerebral ischemia/reperfusion models [10,11]. Emerging evidence suggests its neuroprotection may involve endocannabinoid system modulation, particularly through elevated anandamide (AEA) and 2-arachidonoylglycerol (2-AG) levels [12,13]. This is clinically relevant given the established neuroprotective effects of cannabinoid receptor activation in ischemic injury [14-16]. Cannabinoid type 1 (CB1) receptor and cannabinoid type 2 (CB2) receptor are the two main cannabinoid receptors. In brain tissue, the CB1 receptor is expressed in neurons and astrocytes, whereas the CB2 receptor is expressed in microglial cells and astrocytes. However, whether cannabinoid receptors mediate propofol-induced neuroprotection against brain ischemic injury remains unclear.

Our study employs a dual-model approach to evaluate propofol’s mechanisms: an in vivo rat middle cerebral artery occlusion (MCAO) model and an in vitro oxygen-glucose deprivation/reoxygenation (OGD/R) model using primary neurons [17,18]. We hypothesize that propofol exerts its cerebroprotective effects through CB1 receptor-mediated pathways during ischemia-reperfusion injury.

## Materials and methods

Animals

Fifty-six male Sprague-Dawley rats (body weight 200-300 g) and seven time-dated pregnant dams were sourced from the accredited Experimental Animal Center of the General Hospital of Southern Theatre Command. All experimental protocols adhered to international guidelines for laboratory animal welfare and were specifically approved by the Ethics Committee of the General Hospital of Southern Theatre Command (Guangzhou, Guangdong, China). Fat emulsion injection (C14-24) was purchased from Sichuan Kelun (Sichuan, China).

Experimental design and therapeutic interventions

To systematically evaluate propofol’s neuroprotective ability, 24 SD rats were stratified into three experimental cohorts (n = 8 per group). First, the 24 rats were labeled with consecutive and unique serial numbers ranging from 1 to 24, with each number corresponding to a specific rat. A standardized random number table, a pregenerated table with digits 0-9 arranged in rows and columns commonly used in experimental research, was selected, and a random starting point was set to eliminate subjective bias. Since the total number of rats was a two-digit value, two-digit random numbers (ranging from 00 to 99) were used for the selection process. A consistent direction was predefined to read the random number table. Starting from the predefined point, two-digit random numbers were read in the set direction and filtered according to the following rules: numbers within the range of 01-24 were retained (as rat serial numbers were 1-24; numbers 00 or greater than 24 were discarded), and duplicate numbers were discarded to prevent a single rat from being assigned to multiple groups. Extraction and screening continued until 24 valid random numbers were collected, each corresponding to one rat’s serial number. Once obtained, the 24 valid numbers were sorted in ascending order, and rats were assigned to the three groups according to the sorted sequence. Rats corresponding to the first to eighth smallest valid numbers underwent a 90-minute surgical intervention without vascular occlusion, followed by 24-hour recovery under standard housing conditions (sham group). Rats corresponding to the ninth- to 16th smallest valid numbers were subjected to 90-minute MCAO with subsequent 24-hour reperfusion (MCAO group). Rats corresponding to the 17th to 24th smallest valid numbers received an IV bolus of propofol (50 mg/kg, Fresenius Kabi, China) immediately post-occlusion prior to 24-hour reperfusion (Prop+MCAO group). Terminal assessments included quantitative analysis of cerebral infarction volume using TTC staining, standardized neurofunctional evaluation via modified Bederson scoring, and serum endocannabinoid quantification through liquid chromatography-tandem mass spectrometry (LC-MS/MS). Mortality was recorded during modeling and reperfusion, with total mortality rates of 20.0% and 11.1% in the MCAO and Prop+MCAO groups, respectively, whereas no deaths occurred in the sham-operated group. Rats that died or failed to establish a successful model were excluded, and only surviving rats with successful MCAO were included in subsequent statistical analyses.

**Figure 1:**
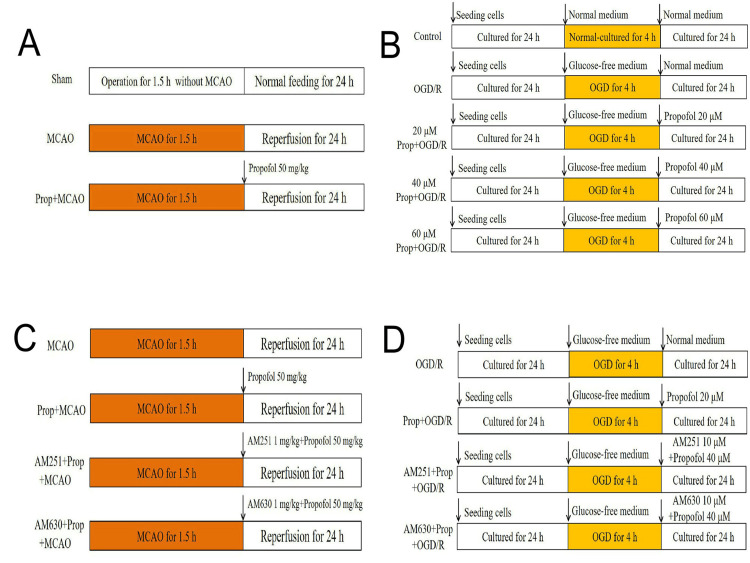
Experimental protocol (A) Impact of propofol on rats with MCAO. SD rats were evenly divided into three groups (n = 8 per group): the sham operation group, the MCAO group, and the propofol+MCAO group. Following treatment, brain infarction volume and neurological deficit scores were measured. Additionally, serum levels of endocannabinoids, AEA and 2-AG, were determined. (B) Influence of propofol on primary-cultured neurons undergoing OGD/R injury. Primary-cultured neurons were categorized into five groups: the normal-culture control group, the OGD/R-affected group, and three propofol-treated groups with different concentrations. After the experimental treatments, multiple indices were evaluated, including cell viability, LDH release, and the cell apoptosis rate. Furthermore, AEA and 2-AG concentrations in the culture medium were measured. (C) Effects of cannabinoid receptor antagonists on propofol-mediated neuroprotection in MCAO rats. SD rats were allocated into four groups (n = 8 per group): the MCAO group, the Prop+MCAO group, the CB1 receptor antagonist AM251+Prop+MCAO group, and the CB2 receptor antagonist AM630+Prop+MCAO group. After treatments, key parameters in ischemic brain tissue were measured, including apoptosis level, neurological score, and the balance between oxidants and antioxidants. (D) Effects of cannabinoid receptor antagonists on propofol-induced cytoprotection in neurons with OGD/R injury. Neurons were divided into four groups: the OGD/R group, the 40 μM propofol-treated group, the AM251+Prop+OGD/R group, and the AM630+Prop+OGD/R group. Following experimental procedures, various cellular indices were analyzed. 2-AG, 2-arachidonoylglycerol; AEA, anandamide; CB1, cannabinoid type 1; LDH, lactate dehydrogenase; MCAO, middle cerebral artery occlusion; OGD/R, oxygen-glucose deprivation/reoxygenation

Similarly, 32 SD rats were assigned into four groups, including MCAO, Prop+MCAO, CB1 antagonist AM251+Prop+MCAO, and CB2 antagonist AM630+Prop+MCAO, with eight rats per group. Rats in the AM251+Prop+MCAO group received an IV bolus of propofol (50 mg/kg) plus 1 mg/kg AM251 immediately post-occlusion prior to 24-hour reperfusion, while rats in the AM630+Prop+MCAO group received propofol (50 mg/kg) plus 1 mg/kg AM630 immediately post-occlusion and prior to 24-hour reperfusion. Treatments for the MCAO and Prop+MCAO groups were as described above. In groups not receiving propofol, an equivalent volume of fat emulsion injection was administered to control for potential effects of the solvent. After the treatments, neuronal apoptotic cell counting, neurological function, and brain tissue oxidant/antioxidant levels were evaluated (Figure 1).

MCAO model

MCAO was implemented to simulate cerebral ischemia/reperfusion pathophysiology. Following established protocols [19], Sprague-Dawley rats received intraperitoneal anesthesia with 2% pentobarbital sodium (60 mg/kg), with core body temperature maintained at 36.5-37.5 °C using a feedback-regulated heating pad throughout the surgical procedures. Transient cerebral ischemia was induced for 90 minutes via intraluminal filament placement, followed by reperfusion achieved through filament withdrawal. Real-time cerebral perfusion monitoring was performed using laser Doppler flowmetry (ALF21, Advance Co., Tokyo, Japan), and inclusion criteria required >80% reduction in cortical blood flow during occlusion and >80% reperfusion recovery relative to baseline values. Only animals meeting these hemodynamic parameters were included in the final data analysis. Subsequently, rats received an i.p. injection of 100 mg/kg pentobarbital sodium to achieve deep anesthesia and were sacrificed by decapitation using a calibrated rodent guillotine. The skull was promptly opened, and the whole brain was carefully isolated and harvested on an ice-cold plate for further processing. Death was confirmed by the complete cessation of respiration and heartbeat.

Infarct volume assessment

Cerebral infarction was quantified using 2,3,5-triphenyltetrazolium chloride (TTC, CAT# 17779, Sigma-Aldrich, St. Louis, Missouri, USA) histochemical staining. Following intraperitoneal administration of 2% pentobarbital (60 mg/kg) for anesthesia, rats were rapidly decapitated. Brains were excised, flash-frozen, and coronally sectioned into 1-mm slices using a cryostat. Tissue sections were immersed in 2% TTC solution (37 °C, 30 minutes) to visualize mitochondrial dehydrogenase activity, followed by fixation in 4% paraformaldehyde (one hour, room temperature). Digital morphometric analysis (Image-Pro Plus 7.0, Media Cybernetics, Inc., Rockville, Maryland, USA) differentiated viable tissue (crimson) from infarct zones (pale). Infarct volume (%) was calculated as:

$\text{Infarct volume (\%)} = \frac{\text{Contralateral hemisphere volume} - \text{Ipsilateral viable volume}}{\text{Contralateral hemisphere volume}} \times 100\%$

Endocannabinoid quantification via LC-MS/MS

Biological specimens (serum and culture media) were cryopreserved at -80 °C following experimental interventions. Samples were processed via protein precipitation using ice-cold methanol/Tris buffer (50 mM, pH 8.0; 1:3 v/v), followed by centrifugation (15,000 × g, 15 min, 4 °C). The resultant supernatant underwent liquid-liquid extraction with chloroform:methanol (2:1 v/v), followed by washing in pure chloroform. Organic phases were evaporated under a nitrogen stream and stored at -80 °C until LC-MS/MS analysis, with methanol reconstitution prior to injection. Quantification of AEA and 2-AG was performed using validated isotope-dilution LC-MS/MS methodology [20]. Analytical performance criteria included intra-assay precision <15% CV, accuracy 85-115% recovery, detection limits 0.1 pmol/L (AEA) and 0.6 pmol/L (2-AG), and minimal matrix-induced ion suppression (<20%). Chromatographic separation employed an ACQUITY UPLC H-Class system (Waters Corporation, Milford, MA, USA) coupled to a TSQ Quantum Triple Quadrupole mass spectrometer (Thermo Scientific, Waltham, Massachusetts, USA). A reversed-phase Athena C18-WP column (50 × 2.1 mm, 3 μm; Waters Corporation) maintained at 40 °C facilitated compound resolution under gradient elution.

Primary cortical neuron isolation and culture

Cortical tissues were aseptically harvested from E16-E18 Sprague-Dawley rat embryos following maternal anesthesia with 2% pentobarbital sodium (60 mg/kg, i.p.). After removal of meninges and vascular components, cerebral cortices were mechanically dissociated into 1 mm³ fragments. Enzymatic digestion employed 0.25% trypsin (Roche #9002-07-7, Basel, Switzerland) in HBSS for 30 minutes at 37 °C, followed by mechanical trituration using fire-polished Pasteur pipettes. Cells were plated on poly-L-lysine-coated surfaces (100 μg/mL) at 5 × 10⁵ cells/cm² in neurobasal medium supplemented with 2% B27 serum-free supplement (v/v, Gibco #17504-44, Waltham, Massachusetts, USA), 0.5 mM L-glutamine, penicillin/streptomycin (100 U/mL), and glucose-free formulation (Gibco #11966-025, USA) for hypoxic exposure. Cultures were maintained at 37 °C in a humidified 5% CO₂ atmosphere, with medium replacements scheduled as follows: a full medium exchange 8 hours post-plating, followed by 50% medium changes every 48 hours. After seven to nine days in vitro, neuronal cultures were subjected to controlled hypoxia (95% N₂/5% CO₂, 37 °C) for four hours in glucose-depleted medium, followed by 24-hour normoxic recovery in complete neurobasal medium to simulate reoxygenation.

Cell viability assessment

Primary neurons were plated in 96-well plates at 5 × 10⁴ cells/well. Following experimental interventions, 20 μL of MTT reagent (5 mg/mL in PBS; Sigma-Aldrich #298-93-1) was added per well. After four-hour incubation (37 °C, 5% CO₂), medium was aspirated and replaced with 150 μL DMSO per well. Plates were shaken (15 minutes, 200 rpm) to solubilize formazan crystals, and optical density was measured at 490 nm using a Tecan Infinite series microplate reader (Tecan Group Ltd., Männedorf, Switzerland). Blank controls containing 150 μL deionized water were processed in parallel to normalize background absorbance.

Lactate dehydrogenase (LDH) activity assay

Neuronal cultures were established in 24-well plates at 5 × 10⁵ cells/well. Following experimental interventions, culture supernatants were collected and clarified through centrifugation (3,000 × g, 10 min). Reaction mixtures containing 150 μL supernatant, 250 μL assay buffer, and 50 μL coenzyme solution were prepared in triplicate. The enzymatic reaction proceeded through two sequential phases: primary incubation at 37 °C for 15 minutes under standard atmospheric conditions and chromogen development with the addition of 250 μL 2,4-dinitrophenylhydrazine followed by dark incubation at 37 °C for 15 minutes. Reactions were terminated with 2.5 mL NaOH (0.4 M), and spectrophotometric measurements (wavelength = 440 nm) were conducted after five minutes of stabilization using a Tecan Spark multimode reader (Tecan Group Ltd). LDH activity quantification followed established protocols [21], with data normalized to total cellular protein content.

Flow cytometric analysis of neuronal apoptosis

Neuronal cultures were established in six-well plates at 2 × 10⁵ cells/well. Post-treatment, cellular suspensions containing both adherent and detached cells were collected through centrifugation (500 × g, five minutes, 4 °C). Cell pellets were washed twice with ice-cold PBS before resuspension in 1× annexin-binding buffer (1 × 10⁶ cells/mL). Staining cocktails containing 5 μL FITC-conjugated annexin V and 2 μL propidium iodide (1.5 μg/mL) were incubated with 100 μL cell suspension for 15 minutes at 25 °C in the dark. Quantitative analysis of apoptotic populations was performed using a BD FACSCanto II flow cytometer (BD Biosciences, San Jose, California, USA) with FACSDiva software (v6.1.3).

Quantification of cerebral apoptosis via TUNEL staining

Cerebral ischemic penumbra sections obtained 24 hours post-reperfusion underwent terminal deoxynucleotidyl transferase dUTP nick-end labeling (TUNEL) analysis following established protocols [19]. Fluorescent microscopy (Nikon Eclipse Ni-E, Nikon Corporation, Tokyo, Japan) captured five random fields per section at 400× magnification. Apoptotic nuclei were quantified using ImageJ software (v1.53k), with cellular density normalized to tissue area (cells/mm²).

Neurological score

Neurological assessment in MCAO rats was conducted using the Bederson scoring system at 24 hours post-reperfusion according to a previous report [22]. The evaluation protocol employed a 5-grade neurological deficit scale: Grade 0 indicated normal neurological function; Grade 1 demonstrated partial forelimb extension impairment on the affected side; Grade 2 manifested as ipsiversive circling behavior; Grade 3 revealed complete loss of weight-bearing capacity in the contralateral limbs; and Grade 4 represented severe consciousness depression with inability to perform spontaneous locomotion. All operations during surgery and outcome assessments were performed by blinded investigators under standardized laboratory conditions.

Protein quantification and immunoblotting analysis

Protein concentrations in cellular lysates and tissue homogenates were determined through Bradford assay quantification. Western blot analysis was performed following established protocols from our laboratory [20], with modifications for enhanced reproducibility. Primary antibodies targeting key apoptotic markers and oxidative stress regulators were employed at 1:200 dilutions: Bax (ab32503), Bcl-2 (ab194583), NOX2/gp91phox (ab310337), superoxide dismutase 2 (SOD2; ab68155), along with housekeeping protein β-actin (ab8226) (all from Abcam, Cambridge, UK). Membranes were probed with horseradish peroxidase-conjugated goat anti-rabbit IgG secondary antibodies (CW01035, CWBIO, Beijing, China) following antigen retrieval procedures. Protein bands were visualized using enhanced chemiluminescence detection and quantified through densitometric analysis with Image Lab™ software (Version 6.1, Bio-Rad Laboratories, Hercules, CA, USA).

Cerebral MDA and GSH evaluation

Cerebral ischemic penumbra tissue was used to evaluate malondialdehyde (MDA) and GSH levels using the MDA (D799762, Sangon Biotech, Shanghai, China) and GSH (BC1175, Solarbio, Beijing, China) kits according to the manufacturer’s instructions.

Intracellular ROS and GSH assessment

For ROS measurement, neuronal cultures (5 × 10⁵ cells/well in six-well plates) underwent 24-hour reoxygenation prior to analysis. Cellular oxidative stress was quantified using a fluorometric assay employing 2’,7’-dichlorodihydrofluorescein diacetate (DCF-DA), a nonfluorescent probe that undergoes ROS-dependent oxidation to fluorescent DCF. Cells were loaded with 100 μM DCF-DA in serum-free medium for 20 minutes at 37 °C, protected from light, followed by three successive PBS washes (five minutes each). Fluorescence imaging was conducted using an Olympus FV10i confocal laser microscope (excitation/emission: 480/535 nm; Olympus Corporation, Tokyo, Japan) with randomized field selection. Digital image analysis was performed using Image-Pro Plus^®^ software (v6.3, Media Cybernetics, Inc.) for fluorescence intensity quantification.

Intracellular glutathione (GSH) levels were determined spectrophotometrically using a commercial assay kit (BC1175, Solarbio). Post-treatment, cell lysates were prepared by homogenization in ice-cold kit-provided lysis buffer (Solution #1) followed by centrifugation (25,000 × g, 10 min, 4 °C). Supernatant aliquots (20 μL) were combined with 140 μL chromogenic reagent (Solution #2/3 mixture) in 96-well plates. After two-minute incubation at ambient temperature, absorbance was measured at 412 nm using a microplate reader. GSH concentrations were normalized to total protein content and calculated against a freshly prepared standard curve. All experimental procedures were conducted in triplicate biological replicates.

Statistical analysis

All statistical computations were performed using IBM SPSS Statistics for Windows, version 23.0 (released 2013; IBM Corp., Armonk, NY, USA). Parametric assumptions were verified through Kolmogorov-Smirnov normality testing and Levene’s test for equality of variance. Intergroup comparisons of continuous variables were analyzed using one-way ANOVA with post hoc Tukey’s testing for multiple comparisons. Nonparametric neurological score data were evaluated using Kruskal-Wallis tests. Experimental results from three independent biological replicates are presented as mean ± SD. A probability threshold of P < 0.05 established statistical significance for all inferential analyses.

## Results

Propofol mitigates cerebral ischemia/reperfusion damage and elevates circulating endocannabinoid concentrations in rodent models

**Figure 2:**
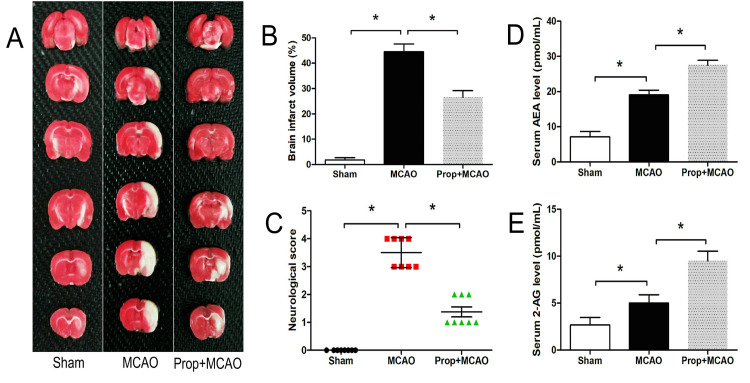
Propofol’s impact on brain ischemic injury and serum cannabinoids in rats (A) TTC staining of rat cerebral slices. (B) Statistical analysis of brain infarct volume in rats. (C) Propofol’s effect on neurological score in rats post-MCAO. (D, E) Propofol-induced increase in serum endocannabinoids AEA and 2-AG. n = 8, * P < 0.05 MCAO, middle cerebral artery occlusion; TTC, triphenyl tetrazolium chloride

As illustrated in Figure 2A-2C, transient MCAO followed by reperfusion induced substantial cerebral infarction and neurological impairment compared to sham-operated controls (P < 0.05). Pharmacological intervention with 50 mg/kg propofol demonstrated significant neuroprotective effects, reducing infarct size (P < 0.05) and improving neurological scores (P < 0.05).

The ischemic challenge elevated serum concentrations of endogenous cannabinoids, with increases in AEA (19.0 ± 1.4 pmol/mL) and 2-AG (5.0 ± 0.9 pmol/mL) relative to baseline measurements (AEA: 7.2 ± 1.5 pmol/mL, 2-AG: 2.7 ± 0.8 pmol/mL; Figure 2D-2E, P < 0.05). Propofol administration potentiated this response, achieving further increases in AEA (27.5 ± 1.3 pmol/mL) and 2-AG (9.5 ± 1.0 pmol/mL) compared with MCAO-only counterparts (P < 0.05). These results collectively demonstrate propofol’s dual capacity to attenuate ischemic brain damage while modulating endocannabinoid levels in rats.

Neuroprotective effects of propofol against OGD/R injury through endocannabinoid modulation

**Figure 3:**
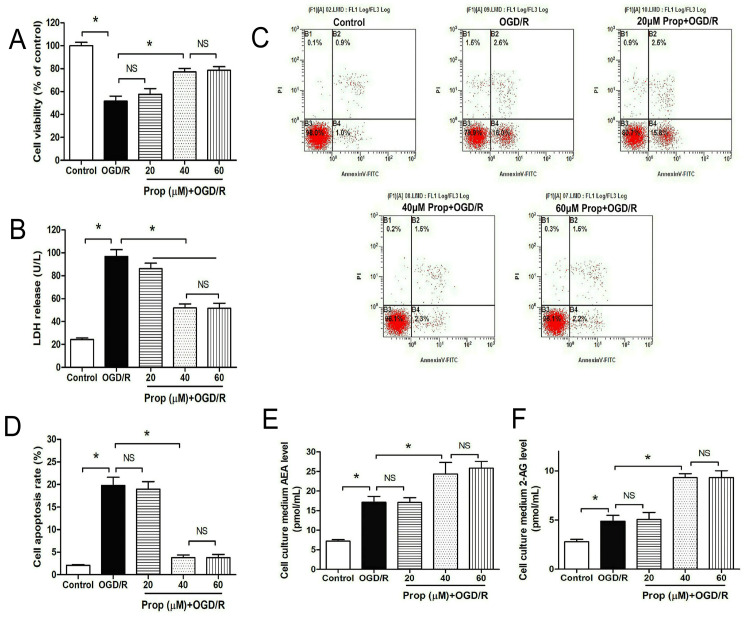
Propofol mitigated OGD/R-induced neuronal injury and augmented cannabinoid levels in the medium (A) Propofol restored cell viability in OGD/R-treated neurons. (B) Propofol reduced LDH release from OGD/R-treated neurons. (C) Flow cytometry results of OGD/R-treated neurons. (D) Propofol decreased the apoptosis rate of OGD/R-treated neurons. (E, F) Propofol increased the levels of cannabinoids AEA and 2-AG in the medium of OGD/R-treated neurons. All experiments were repeated three times, and statistical significance between groups was analyzed using one-way ANOVA with post hoc Tukey’s testing for multiple comparisons. n = 8, * P < 0.05, NS: not significant. 2-AG, 2-arachidonoylglycerol; AEA, anandamide; LDH, lactate dehydrogenase; OGD/R, oxygen-glucose deprivation/reoxygenation

As illustrated in Figure 3A-3D, OGD/R significantly reduced neuronal viability while elevating LDH release and apoptotic rates in primary cultured neurons compared with control conditions (P < 0.05). Notably, propofol administration demonstrated concentration-dependent protective effects: treatment with 40 μM or 60 μM propofol effectively restored cellular viability and attenuated both LDH leakage and apoptosis (P < 0.05), whereas the 20 μM concentration showed no statistically significant neuroprotection against OGD/R injury (P > 0.05).

The analysis of endocannabinoid dynamics revealed concurrent changes (Figure 3E-3F). OGD/R stimulation elevated extracellular levels of AEA (17.1 ± 1.5 pmol/mL) and 2-AG (4.9 ± 0.6 pmol/mL) compared with baseline measurements (AEA: 7.2 ± 1.0 pmol/mL, 2-AG: 2.8 ± 0.6 pmol/mL, P < 0.05). Therapeutic concentrations of propofol (40-60 μM) administered over 24 hours further potentiated these endocannabinoid elevations (40 μM propofol: AEA 24.4 ± 3.0 pmol/mL, 2-AG 9.3 ± 0.4 pmol/mL; 60 μM propofol: AEA 25.9 ± 1.7 pmol/mL, 2-AG 9.3 ± 0.7 pmol/mL) beyond OGD/R-induced levels. The pharmacological effects of a drug are closely related to its dosage. In this study, 20 μM propofol did not produce significant neuroprotection against OGD/R injury or enhance endocannabinoid generation, whereas 40 μM and 60 μM propofol did. Specifically, 20 μM propofol failed to promote endocannabinoid production sufficiently (AEA: 17.1 ± 1.2 pmol/mL, 2-AG: 5.1 ± 0.7 pmol/mL) and thus did not activate cannabinoid receptors. This dose-response relationship between propofol exposure and endocannabinoid production suggests a novel mechanistic pathway through which propofol exerts neuroprotective effects against ischemia-reperfusion injury.

CB1 receptor dependency of propofol’s neuroprotection in cerebral ischemia

**Figure 4:**
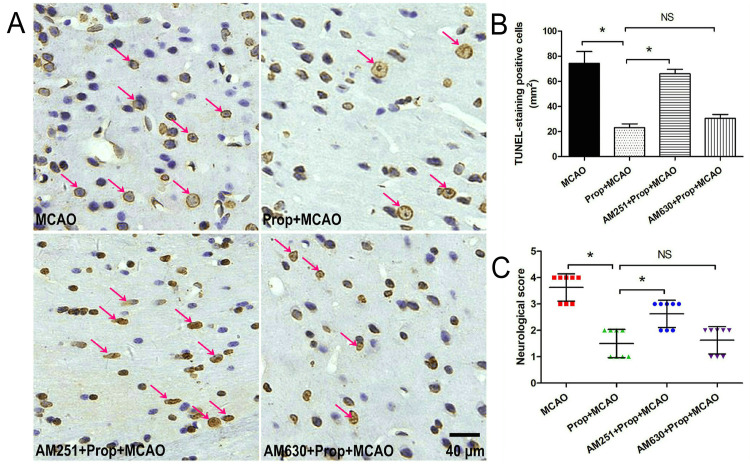
CB1 receptor antagonist reversed propofol-induced neuroprotection in rats with brain I/R injury (A) TUNEL staining outcomes of the brain ischemic area in rats. (B) Statistical analysis of TUNEL-positive cells in the ischemic brain region. (C) CB1 receptor antagonist AM251 reversed propofol-induced improvement in neurological score. n = 8, * P < 0.05, red arrow: TUNEL-positive cells, bar = 40 μm. CB1, cannabinoid type 1; MCAO, middle cerebral artery occlusion; NS, not significant; TUNEL, terminal deoxynucleotidyl transferase dUTP nick-end labeling

As demonstrated in Figure 4A-4C, propofol administration (50 mg/kg) exerted significant cerebroprotective effects in MCAO rats, evidenced by reduced neuronal apoptosis (P < 0.05) and improved neurological outcomes compared with untreated ischemic controls (P < 0.05). Pharmacological intervention studies revealed distinct receptor specificity: pretreatment with the CB1 receptor antagonist AM251 substantially attenuated propofol-mediated anti-apoptotic effects and functional recovery (P < 0.05), whereas CB2 receptor blockade using AM630 did not produce notable alterations in propofol’s therapeutic efficacy (P > 0.05). These findings establish the CB1 receptor as a critical mediator of propofol’s neuroprotective mechanisms against ischemia-reperfusion injury, while functionally distinguishing its role from the CB2 receptor pathway in this therapeutic context. The dose-specific receptor antagonism data suggest that propofol’s anti-apoptotic actions and neurological restoration are preferentially mediated through CB1-dependent signaling cascades.

CB1 receptor mediation of propofol’s cytoprotective effects in OGD/R-injured neurons

**Figure 5:**
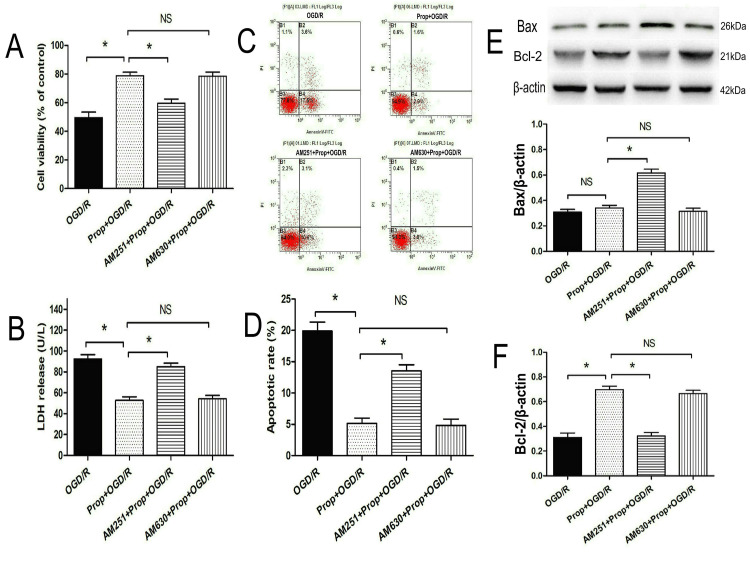
CB1 receptor antagonist reversed propofol-induced neuroprotection in OGD/R neurons (A) CB1 receptor antagonist AM251 counteracted propofol-induced cytoprotection in OGD/R neurons (n = 8). (B) AM251 reversed propofol-induced suppression of LDH release (n = 8). (C, D) AM251 reversed propofol-induced inhibition of cell apoptosis in OGD/R neurons (n = 8). (E) AM251 increased expression of the pro-apoptotic protein Bax in OGD/R neurons (n = 4). (F) AM251 reversed propofol-induced upregulation of the anti-apoptotic protein Bcl-2 in OGD/R neurons (n = 4). All experiments were repeated three times, and significance between groups was analyzed by one-way ANOVA with post hoc Tukey’s testing for multiple comparisons. * P < 0.05. CB1, cannabinoid type 1; LDH, lactate dehydrogenase; NS, not significant; OGD/R, oxygen-glucose deprivation/reoxygenation

As shown in Figure 5A, 5B, propofol (40 μM) significantly attenuated OGD/R-induced cytotoxicity in primary neurons, improving cellular viability (P < 0.05) and reducing LDH release compared with the OGD/R group (P < 0.05). Pharmacological inhibition of the CB1 receptor with AM251 abolished these protective effects (P < 0.05), whereas CB2 receptor antagonism via AM630 did not produce significant interference (P > 0.05), confirming functional specificity in receptor-mediated responses.

Further investigation of apoptotic mechanisms (Figure 5C-5F) revealed that propofol suppressed neuronal apoptosis (P < 0.05) and upregulated the anti-apoptotic protein Bcl-2 compared with the OGD/R group (P < 0.05). This cytoprotective profile was partially reversed by AM251 pretreatment, which concurrently elevated expression of the pro-apoptotic marker Bax compared with the Prop+OGD/R group (P < 0.05). In contrast, AM630 exhibited no modulatory effects on apoptotic indices (P > 0.05).

Collectively, these data demonstrate that propofol’s anti-apoptotic actions against ischemic-reperfusion injury are pharmacologically dependent on CB1 receptor activation, highlighting its role as a critical molecular pathway in neuronal survival under metabolic stress.

CB1 receptor mediates propofol’s redox modulation in cerebral ischemia

**Figure 6:**
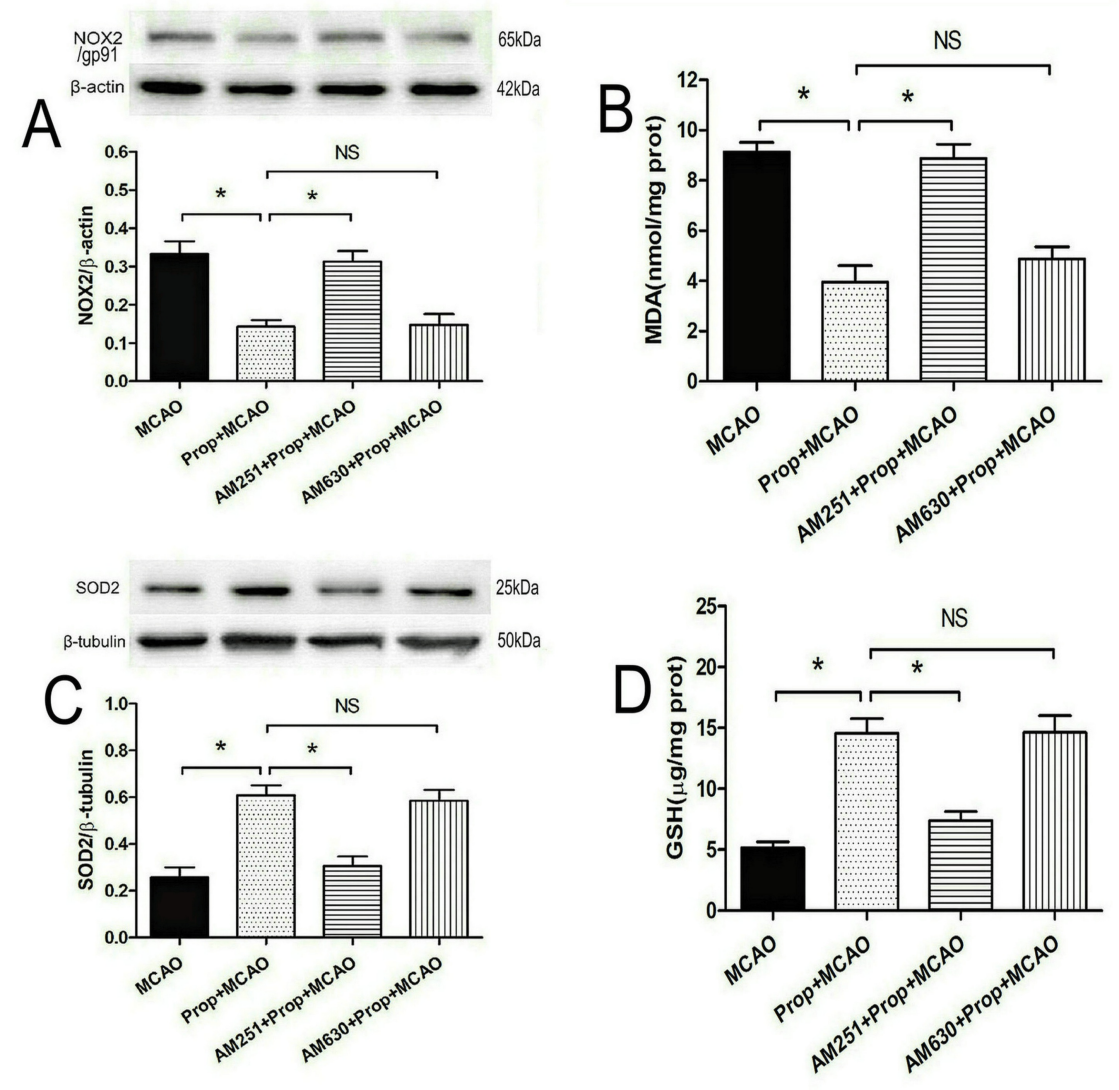
CB1 receptor antagonist reversed propofol-induced antioxidative effects in rats with brain I/R injury (A) CB1 receptor antagonist AM251 abolished propofol-induced downregulation of NOX2 protein (n = 4). (B) AM251 reversed propofol-induced reduction in cerebral MDA levels (n = 8). (C) AM251 reversed propofol-induced upregulation of cerebral SOD2 expression (n = 4). (D) AM251 reversed propofol-induced increase in cerebral GSH levels (n = 8). * P < 0.05, NS: not significant. CB1, cannabinoid type 1; GSH, glutathione; MCAO, middle cerebral artery occlusion; MDA, malondialdehyde; NOX2, NADPH oxidase 2; SOD2, superoxide dismutase 2

As demonstrated in Figure 6A-6D, propofol (50 mg/kg) significantly improved redox homeostasis in MCAO rats by reducing oxidative stress markers, specifically suppressing NADPH oxidase subunit NOX2/gp91 expression (P < 0.05) and lowering MDA levels (P < 0.05), while simultaneously enhancing antioxidant defenses through upregulation of mitochondrial SOD2 and increased GSH levels compared with untreated ischemic controls (P < 0.05). Pharmacological interrogation revealed receptor-specific regulation: CB1 receptor antagonism with AM251 completely abolished propofol’s dual antioxidant effects, restoring NOX2/gp91 and MDA to ischemic baseline levels (P < 0.05) while negating SOD2 and GSH enhancements (P < 0.05). In contrast, CB2 receptor blockade via AM630 showed no significant impact on these redox parameters (P > 0.05), underscoring the selective involvement of CB1 signaling. These results establish CB1 receptors as critical mediators of propofol’s therapeutic modulation of oxidative stress pathways, effectively linking its antioxidant actions with canonical endocannabinoid signaling in ischemic brain tissue.

CB1 receptor mediates propofol’s redox regulation in ischemic neurons

**Figure 7:**
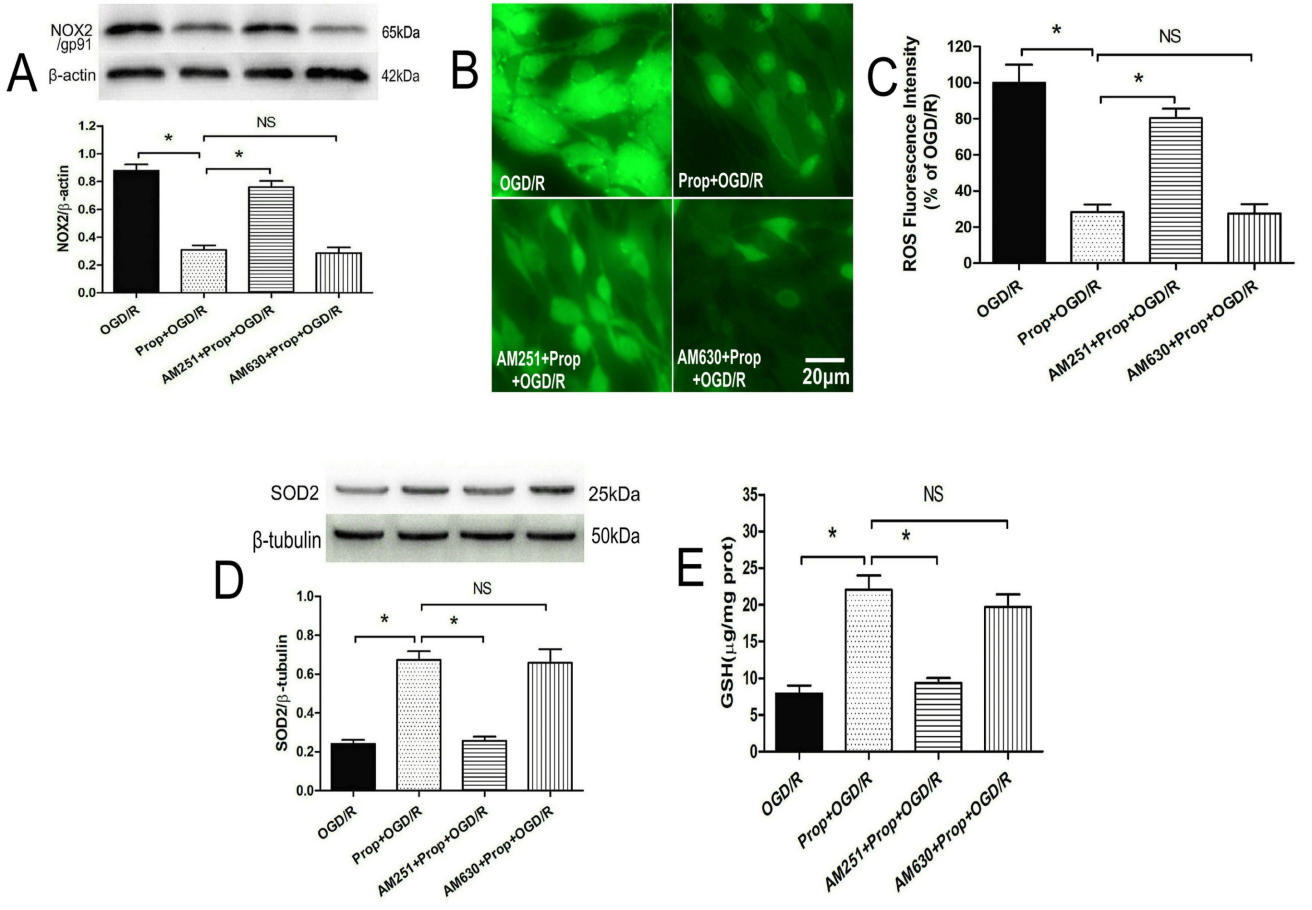
CB1 receptor antagonist reversed propofol-induced antioxidant effects in OGD/R neurons (A) CB1 receptor antagonist AM251 reversed propofol-induced NOX2 protein downregulation (n = 4). (B, C) AM251 reversed propofol-induced intracellular ROS decrease (n = 8). (D) AM251 reversed propofol-induced SOD2 upregulation (n = 4). (E) AM251 reversed propofol-induced intracellular GSH increase (n = 8). All experiments were repeated three times, and statistical significance between groups was analyzed using one-way ANOVA with post hoc Tukey’s testing for multiple comparisons. * P < 0.05, bar = 20 μm. CB1, cannabinoid type 1; GSH, glutathione; NOX2, NADPH oxidase 2; NS, not significant; OGD/R, oxygen-glucose deprivation/reoxygenation; ROS, reactive oxygen species; SOD2, superoxide dismutase 2

As shown in Figure 7A-7E, propofol (40 μM) exerted dual antioxidant effects in OGD/R-challenged neurons, suppressing oxidative stress markers through reduction in NOX2/gp91 protein expression (P < 0.05) and decreased ROS levels (P < 0.05), while concurrently enhancing antioxidant capacity via upregulation of SOD2 and increased GSH levels compared with untreated OGD/R controls (P < 0.05). Pharmacological dissection of receptor involvement revealed selective pathway regulation: CB1 receptor blockade with AM251 completely abolished propofol’s redox-modulating effects, restoring NOX2/gp91 and ROS to pathological levels (P < 0.05) while reversing SOD2 and GSH enhancements to baseline (P < 0.05). In contrast, CB2 receptor antagonism via AM630 showed no significant interference with propofol’s antioxidant efficacy (P > 0.05), demonstrating pathway specificity. These data mechanistically link CB1 receptor activation to propofol’s ability to restore neuronal redox equilibrium under ischemic conditions, positioning it as a critical regulator of mitochondrial antioxidant pathways in neurodegenerative cascades.

## Discussion

This study systematically evaluated propofol’s cerebroprotective mechanisms across in vivo and in vitro ischemia models. In rodent MCAO experiments, IV propofol (50 mg/kg) demonstrated multi-modal protective effects, including reduction in cerebral infarct area, decrease in apoptotic indices, and significant mitigation of neurological deficits compared with untreated ischemic controls. Concurrently, propofol administration modulated redox homeostasis by suppressing oxidative markers (NOX2/gp91 and MDA) while enhancing antioxidant defenses (SOD2 and GSH). Serum analysis revealed elevated endocannabinoid levels (AEA and 2-AG), suggesting systemic neuromodulatory effects.

**Figure 8:**
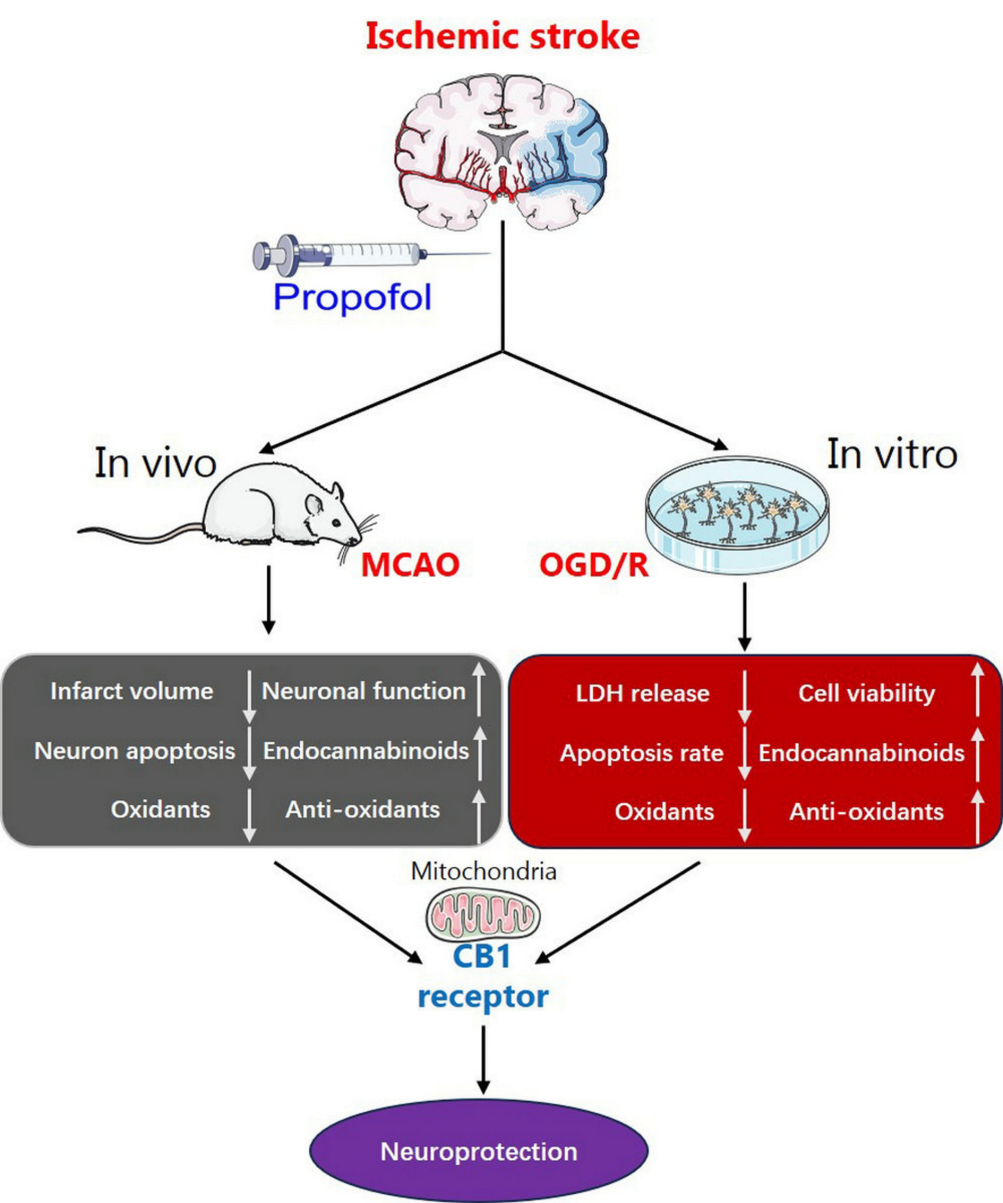
Schematic diagram illustrating the neuroprotective effects of propofol in ischemic brain injury via CB1 receptor-mediated pathways In in vivo experiments, propofol administration led to a reduction in brain infarct volume, a decrease in the number of apoptotic neurons, and a decline in intracerebral oxidant levels, including NOX2/gp91 and MDA. Moreover, it restored neuronal function, boosted brain endocannabinoid levels, and increased antioxidant concentrations. In in vitro studies, propofol exposure resulted in decreased LDH release, a lower cell apoptosis rate, and reduced intracellular oxidant levels, including NOX2/gp91 and ROS. Simultaneously, propofol restored cell viability and intracellular antioxidant levels (SOD2 and GSH) and increased AEA and 2-AG levels in the culture medium. Notably, the neuroprotective effect of propofol against brain ischemia was reversed by a CB1 receptor antagonist, indicating that CB1 receptor activation mediates propofol’s neuroprotective effects in ischemic brain injury. 2-AG, 2-arachidonoylglycerol; AEA, anandamide; CB1, cannabinoid type 1; GSH, glutathione; LDH, lactate dehydrogenase; MDA, malondialdehyde; MCAO, middle cerebral artery occlusion; NOX2, NADPH oxidase 2; OGD/R, oxygen-glucose deprivation/reoxygenation; SOD2, superoxide dismutase 2 This figure was created using images from SMART - Servier Medical ART (smart.servier.com), licensed under Creative Commons Attribution 4.0 International (CC BY 4.0), and assembled in Microsoft PowerPoint (Microsoft Corporation, Redmond, WA, USA).

In neuronal OGD/R models, propofol (40 μM) exhibited concentration-dependent cytoprotection, reducing LDH leakage and apoptosis rates while restoring membrane integrity (cell viability). Intracellular redox balance was improved through ROS suppression and antioxidant upregulation (SOD2 and GSH), accompanied by elevated Bcl-2 expression and extracellular endocannabinoid accumulation (AEA and 2-AG). Pharmacological validation confirmed receptor specificity: CB1 antagonism with AM251 completely abolished propofol’s protective effects across models, whereas CB2 blockade via AM630 showed no significant interference. These findings establish CB1 receptor activation as the principal mechanism underlying propofol’s therapeutic efficacy against ischemic neurodegeneration (Figure 8).

Ischemic stroke remains a global health challenge, imposing significant socioeconomic burdens on affected families and healthcare systems. Current therapeutic limitations stem from two primary factors: (1) the restricted regenerative capacity of ischemic neurons, which leads to prolonged neurological recovery [23,24], and (2) the narrow therapeutic window (≤4.5 hours post-onset) of r-TPA, which restricts treatment availability to fewer than 10% of patients [1]. These constraints underscore the critical need for novel therapeutic interventions. The pathophysiology of cerebral ischemia/reperfusion injury involves paradoxical oxidative damage during blood flow restoration. This oxidative cascade depletes endogenous antioxidants, disrupts cellular integrity, and ultimately contributes to neuronal apoptosis and functional impairment [25-27]. Consequently, antioxidant therapies represent a promising therapeutic strategy.

Propofol, a widely used IV anesthetic, demonstrates neuroprotective properties in cerebral ischemia. Mechanistic studies suggest that its efficacy may derive from dual antioxidant and anti-inflammatory actions [11,28]. Emerging evidence indicates that propofol modulates endocannabinoid signaling, with reports of increased AEA and 2-AG levels in both in vivo and in vitro models [12,13]. Notably, cannabinoid receptor activation has shown neuroprotective potential in cerebral ischemia [16,29], prompting our investigation into endocannabinoid-mediated mechanisms underlying propofol’s neuroprotection. Our experimental findings demonstrate that propofol administration elevated AEA and 2-AG levels in rat serum and neuronal culture medium, reduced cerebral infarct volume, improved neurological outcomes in MCAO models, and attenuated OGD/R-induced neuronal apoptosis in vitro.

Previous work established CB1 receptor-mediated neuroprotection through reduction of glutamate excitotoxicity and oxidative stress [19], contrasting with the CB2 receptor’s predominant role in neuroinflammation modulation. Current results show that CB1 antagonist AM251, but not CB2 antagonist AM630, significantly reversed propofol’s protective effects, confirming CB1 receptor dependency. Post-reperfusion oxidative pathophysiology involves NOX2-mediated ROS generation and lipid peroxidation markers such as MDA [30]. Our data reveal propofol’s antioxidant mechanism through NOX2 downregulation, ROS reduction, and enhanced SOD2 and GSH levels. These antioxidant effects were specifically abrogated by CB1 receptor blockade, aligning with previous findings of CB1-mediated oxidative homeostasis regulation [31-33].

SOD2 is an antioxidant enzyme located in mitochondria, and NOX2 is an enzyme that produces ROS in the plasma membrane. We previously reported that CB1 receptor upregulation can increase mitochondrial SOD2 levels and protect neurons against oxidative stress injury [31]. Moreover, in the present investigation, we found that CB1 receptor antagonist AM251 abolished propofol-induced neuronal SOD2 upregulation, NOX2 downregulation, and ROS accumulation. These findings indicate that the anesthetic propofol protects OGD/R-induced brain injury by increasing mitochondrial SOD2 levels, which then inhibits intracellular NOX2 and ROS production.

Currently, based on the findings of multiple studies [34,35], the neuronal CB1 receptor is known to be expressed in both mitochondria and the cell membrane. In one of our prior research projects [30], we speculated that the mitochondrial CB1 receptor, rather than the CB1 receptor expressed on the cell membrane, mediates the neuroprotective effect of the cannabinoid CB1 agonist ACEA against brain ischemia. In light of this, as depicted in Figure 8 of this investigation, we postulate that the mitochondrial CB1 receptor also plays a role in mediating the neuroprotection conferred by propofol against brain ischemia/reperfusion injury. This hypothesis is based on the understanding that the mitochondrial CB1 receptor has a distinct function in the context of neuroprotection, as demonstrated in our previous work on ACEA, and we believe it may act similarly in the case of propofol’s effects on brain ischemia/reperfusion injury. The observed neuroprotection parallels propofol’s documented organoprotective effects in peripheral ischemia models [36-38], suggesting potential broader applications of this mechanism.

Current understanding of propofol’s endocannabinoid modulation primarily centers on its inhibition of fatty acid amide hydrolase (FAAH), the principal enzyme responsible for endocannabinoid degradation. This enzymatic suppression distinguishes propofol from other IV anesthetics (thiopental, etomidate, and midazolam), which demonstrate no significant FAAH inhibitory capacity in experimental models [12]. The resultant elevation of AEA and 2-AG levels likely activates cannabinoid receptor signaling pathways, potentially mediating organoprotective effects against ischemia/reperfusion injury.

However, several critical knowledge gaps require attention. Although experimental evidence confirms endocannabinoid elevation in rodent models and cell cultures, the pharmacokinetic and pharmacodynamic effects of propofol on human endocannabinoid physiology remain uncharacterized. To address this, future clinical investigations should quantify serum and CSF endocannabinoid concentrations following therapeutic propofol administration, enabling translation from experimental models to clinical practice. Our experimental paradigm focused on acute-phase neuroprotection (≤72 hours post-ischemia), consistent with current stroke intervention timelines. Nevertheless, the potential therapeutic efficacy of propofol during delayed phases (≥3 days post-ischemia), when secondary neurodegeneration and repair mechanisms dominate, warrants systematic evaluation through extended observation studies. In the future, a clinical trial is needed to verify whether propofol provides neuroprotection against brain ischemia in stroke patients. Moreover, subsequent investigations should examine whether propofol can increase endocannabinoid levels in the CSF of patients receiving external ventricular drainage placement.

Notably, while the FAAH inhibition hypothesis provides a plausible mechanism, alternative pathways for propofol’s endocannabinoid modulation cannot be excluded. Recent proteomic studies suggest potential drug-protein interactions that might influence endocannabinoid biosynthesis or transport mechanisms [39]. Furthermore, the tissue specificity of these effects across different organ systems remains underexplored. In this study, we found that propofol administration reduced brain ischemia-induced oxidative stress injury in rats, and the limited therapeutic time window is due to cerebral ischemia/reperfusion-induced oxidative injury. Therefore, these findings may prolong the therapeutic time window for stroke. Moreover, these findings position CB1 receptor activation as the principal molecular mechanism underlying propofol’s neurovascular protection, offering novel insights into targeted therapeutic strategies for ischemic stroke.

However, there are four limitations in this study. First, in this investigation, only the CB1 antagonist AM251 was used to explore the role of the CB1 receptor in propofol-induced neuroprotection. The use of a CB1 antagonist can only demonstrate that the CB1 receptor mediates propofol-induced neuroprotection pharmacologically; CB1-siRNA and CB1-knockout mice are needed to prove the role of CB1 genetically. Second, the results of this study showed that the CB1 receptor mediates propofol-induced neuroprotection against brain ischemic injury in the acute phase (≤72 hours post-ischemia); however, in the delayed phase (≥3 days post-ischemia), it is unknown which cannabinoid receptor mediates propofol’s neuroprotection. Third, the findings of this study are from in vivo and in vitro experiments, so clinical trials are needed to verify these results. Fourth, this study has a relatively small sample size, which limits statistical power and may introduce potential bias.

## Conclusions

The findings of this investigation demonstrate that propofol induces neuroprotection and produces endocannabinoids (AEA and 2-AG) in vivo and in vitro. In vivo, propofol (50 mg/kg) protects ischemic brain tissue in rats, including reducing cerebral infarct area and apoptotic neurons, improving neurological outcomes, and decreasing oxidative stress markers. Concurrently, propofol administration elevated antioxidant defenses through enhanced SOD2 activity and increased GSH production. In vitro, propofol (40 μM) reduces MCAO-induced neuronal injury by decreasing ROS generation and restoring antioxidant levels. However, the CB1 receptor antagonist AM251, but not the CB2 receptor antagonist AM630, abolishes propofol’s protective effects in both in vivo and in vitro experiments. These findings establish that the CB1 receptor mediates propofol’s neuroprotective activity against ischemic cerebral damage.
